# *Ganoderma lucidum* Polysaccharide Enzymatic Hydrolysate Suppresses the Growth of Human Colon Cancer Cells via Inducing Apoptosis

**DOI:** 10.1177/0963689720931435

**Published:** 2020-06-04

**Authors:** Jing hui Bai, Jian Xu, Jian Zhao, Rui Zhang

**Affiliations:** 1 Department of Internal Medicine, Cancer Hospital of China Medical University, Liaoning Cancer Hospital & Institute, Shenyang, China; 2 Department of Colorectal Surgery, Cancer Hospital of China Medical University, Liaoning Cancer Hospital & Institute, Shenyang, China

**Keywords:** colon cancer, enzymatically hydrolyzed polysaccharide, *Ganoderma lucidum*

## Abstract

*Ganoderma lucidum* is a popular traditional Chinese medicine used in China to improve health. Previous researches have revealed that the polysaccharide from *G. lucidum* could exert diversity activities, including immunomodulation, antioxidant, and antitumor effects. However, the effect of enzymatically hydrolyzed *G. lucidum* polysaccharide (EGLP) in colorectal cancer (CRC) progression remains unknown. The present research aimed to investigate the antitumor mechanism of EGLP in human colon cancer cells. For this purpose, the cytotoxic effects of EGLP were measured by the (3-(4,5)-dimethylthiahiazo (-z-y1)-3,5-di-phenytetrazoliumromide (MTT) method. The apoptosis was evoked upon EGLP treatment, which was assayed using flow cytometry. The results indicated that EGLP may induce apoptosis in human colon cancer cell (HCT-116) cells via the upregulation of BCL-2 associated X protein (Bax), phospho-extracellular regulated protein kinases (P-ERK), and cleaved caspase-3 expression and downregulation of B-cell lymphoma-2 (Bcl-2), phospho-serine/threonine kinase 1 (p-Akt1), and cyclo-oxygen-ase (COX-2) expression. The obtained findings indicated EGLP as a new therapeutic agent in fighting CRC.

## Introduction

Colorectal cancer (CRC) is the third most deadly cancer for men and women and the third most frequently diagnosed cancer across the world^1^. This prevalence has turned CRC into a major health problem across the world, and its incidence is promptly rising in China. Although research continues for more effective therapies, surgery, chemotherapy, and radiotherapy are the most commonly used treatments of CRC. However, organ toxicity, eventual resistance, and limited effectiveness remain serious problems^2^. Thus, novel therapeutic strategies are urgently required to assuage those side effects and enhance antitumor activity associated with this malignancy. However, it is well established that lifestyles and environmental factors, including physical inactivity, smoking, diet low in fruits or vegetables along with diet rich in red meat and saturated fat, are involved in the development of CRC^3,4^. In addition, many researches imply that diet habits play an important role in CRC carcinogenesis^5^. Thus, dietary regulation by consumption of natural nutrients or pharmacological agents with cancer chemopreventive effects could decrease the incidence of CRC^6^. Natural polysaccharides are the main biologically active components in natural medicine^7^.

*Ganoderma lucidum* is a popular fungus and has been widely used as a traditional Chinese medicine in China for the prevention and treatment of a variety of diseases. Polysaccharides are the main active components of *G. lucidum* and exert a wide range of pharmacological activities, including immunomodulation, anticolon carcinoma, and antioxidation effects^8,9^. The pharmacological activities of polysaccharides are closely related to their molecular structures, including sulfate content, viscosity, average molecular weight, and monosaccharide proportion^10^. High molecular weights, structural complexity, and viscous properties of crude polysaccharides may hinder their clinical application, particularly as therapeutic agents^11^. Low molecular weight polysaccharide could overcome these problems. Previous researches have reported that enzymatically degraded polysaccharide possesses higher antioxidative, antimelanogenesis, and moisture-preserving activities than the crude polysaccharide^10,12^. Besides, increasing evidence has shown that low molecular weight polysaccharide exerts anticancer effects toward different tumors, such as ovarian cancer, colon cancer, and prostate cancer^13^. Thus, the production of low molecular weight oligosaccharide from *G. lucidum* is necessary to improve its biological activity.

According to the literature, there are several chemical and physical ways to hydrolyze the crude polysaccharide, such as oxidative degradation, ultrasonic degradation, and enzymatic degradation^14,15^. Among them, enzymatic degradation is an effective way for high selectivity and substrate specificity, which could produce a well-defined structure of polysaccharide^10^. As far as we know, little research on the anticolon cancer effect of the enzymatically hydrolyzed *G. lucidum* polysaccharide (EGLP) has been conducted. In the present research, the effect of EGLP on colorectal cancer cell apoptosis in vitro was further investigated and the underlying mechanism of its action was investigated.

## Materials and Methods

### Materials and Chemicals

The fruiting body of *G. lucidum* was procured from Xunwu Hengshun biotechnology company (Ganzhou, Jiangxi, China) and was identified as an artificial cultivar. Cellulase was purchased from Sigma-Aldrich (St. Louis, MO, USA). All other solvents and chemicals used were procured from Aladdin (Shanghai, China).

### Extraction and Separation of Crude *G. lucidum* Polysaccharide

*G. lucidum* polysaccharide (GLP) was extracted from the fruiting body of *G. lucidum* by hot water extraction based on previous literature with minor modification^16^. Briefly, the extraction parameters were extraction temperature of 95°C, extraction time of 2 h, and water/solid of 12:1. After the extract was centrifuged, the supernatant was collected and condensed at 60°C under vacuum, and then added into four volumes of 70% ethanol at 2°C for 12 h to precipitate the polysaccharide. To remove the proteins, the precipitates were deproteinated by Sevag method^17^. Then the crude polysaccharide was further purified with dialysis membrane (cutoff 10 kDa) against pure water. Finally, the high molecular weight crude polysaccharide was dried by lyophilization and named GLP.

### Preparation of GLP-Degraded Fragments

GLP was enzymatically degraded by cellulase for 2 h under the condition of pH 5.5 and 50°C. The hydrolysate was dialyzed with dialysis membrane (cutoff 10 kDa) against pure water. The nondialysate was collected and dried by lyophilization. The degradation fragment from hydrolysates of 2 h was named EGLP. The degradation fragment was further purified with the dialysis membrane (cutoff 10 kDa) against pure water. Finally, EGLP was dried by lyophilization. The content of carbohydrate was measured by the phenol–sulfuric acid method. Sulfate content was measured by barium chloride–gelatin method. Protein content was measured by the Bradford method. Uronic acid content was measured by uronic acid carbazole method.

### Molecular Weight and Homogeneity Analysis by SEC-MALLS-RI Measurement

The molecular weight and homogeneity of GLP/EGLP were assayed by size exclusion chromatography combined with multiangle laser light scattering detector and refractive index detector (SEC-MALLS-RI, Waters 2695, USA). Shodex-OHpak SB-804 HQ column (Shodex, Japan) (8.0 mm × 300 mm) was used as the separation medium. The mobile phase was 0.1 M sodium chloride at a flow rate of 0.7 ml/min. The column temperature was set at 30°C, all samples were dissolved with the mobile phase, and the injection volume was 0.1 ml (1 mg/ml).

### ^1^H-NMR Analysis

EGLP, 30.0 mg, was weighted and dissolved in D_2_O (0.5 ml). Nuclear magnetic resonance (^1^H-NMR, Zhongke-Niuji) of the EGLP was measured by AS 400 MHz NMR spectrometer (Wuhan, China).

### Cell Lines and Cell Culture

Human colorectal adenocarcinoma HCT-116 and colon epithelial nontumorigenic (CCD18-Co) cell lines were obtained from American Type Culture Collection (ATCC; Manassas, VA, USA). The cells were grown in Roswell Park Memorial Institute (RPMI-1640) medium (Thermo Fisher), and culture media were supplemented with 100 U/ml penicillin, 100 µg/ml streptomycin (Sigma-Aldrich), and 10% fetal bovine serum (Thermo Fisher), and maintained in a humidified atmosphere containing 5% CO_2_ at 37°C.

### Cell Viability Assay

The effect of GLP or EGLP on HCT-116 and CCD18-Co cells survival was measured by MTT assay according to the previous method with some modifications^18^. Briefly, 5 × 10^4^ cells/well of colon cancer or CCD18-Co cells were cultured in a 96-well plate for 24 h with 5% CO_2_ at 37°C. The cells were treated with 150 µg/ml (GLP/EGLP) or an equivalent volume of dimethyl sulfoxide (DMSO) vehicle control for 24, 48, and 72 h. After the indicated incubation time, 20 µl of MTT reagent was added into each well. After incubation for 2 h, the media was removed, and the formed insoluble formazan crystals were dissolved in DMSO and quantified at 540 nm using the microplate reader (BioTek, Winooski, VT, USA). All samples were assayed in quadruplicate and experiments were repeated three times.

### Cell Apoptotic Analysis

The Annexin fluorescein isothiocyanate (V-FITC) apoptosis detection kit (BD Biosciences, Franklin Lakes, NJ, USA) was used to measure HCT-116 cell apoptosis according to the manufacturer’s protocol. Briefly, the HCT-116 cells were seeded in complete culture medium and incubated with EGLP (150 and 300 µg/ml) for 48 h. Then, the HCT-116 cells were collected by centrifugation at 10,000 rpm for 5 min at 4°C and resuspended in 500 µl Annexin V-FITC binding buffer. HCT-116 cells were incubated with 5 µl Annexin V-FITC and 5 µl propidium iodide (PI) in the dark for 15 min. The apoptosis rates were quantitatively analyzed with a FACSCalibur flow cytometer (Beckman Coulter, Brea, CA, USA).

### Western Blot Analysis

HCT-116 cells were collected and lysed in radio immunoprecipitation assay (RIPA) lysis buffer (Sigma-Aldrich) containing protease inhibitors and phosphatase. Protein concentration of the lysates was quantified using the bicinchoninic acid Protein Assay Kit (Jiancheng Bioengineering Institute, Nanjing, Jiangsu, China). The proteins were separated by 10% sodium dodecyl sulfate polyacrylamide gel electrophoresis (SDS-PAGE) at 110 V for 2 h and then transferred onto a polyvinylidene fluoride membrane (Millipore, Boston, MA, USA) at 60 V for 2 h. After blocking with 5% nonfat milk at room temperature for 1 h, the polyvinylidene fluoride membranes were incubated with anti-Bcl-2 (1:1,000; Abcam, Cambridge, UK), anti-Bax (1:1,000; Abcam), anti-p-Akt1 (1:1,000; Abcam), anti-P-ERK (1:1,000; Abcam), and glyceraldehyde 3-phosphate dehydrogenase (GAPDH) (1:1,000; Abcam) primary antibodies overnight at 4°C. The polyvinylidene fluoride membranes were washed with tris buffered saline tween (TBST) and incubated with a horseradish peroxidase–conjugated secondary antibody at room temperature for 45 min. Visualization was performed with a chemiluminescent detection kit (Millipore). The densitometry of the bands was analyzed using Gel-Pro 6.3 software (Media Cybernetics, Inc., Bethesda, MD,USA).

### Quantitative Real-Time Polymerase Chain Reaction (qRT-PCR)

Total ribonucleic acid (RNA) was isolated from HCT-116 cells using Trizol reagent (Takara Bio, Inc., Dalian, Liaoning, China) according to the manufacturer’s protocol. Complementary deoxyribonucleic acid (cDNA) was synthesized from total RNA using the First Strand cDNA Synthesis Kit (TransGen, Beijing, China). RT-PCR was carried out with an SYBR Green qPCR Master Mix kit (Takara Bio, Inc.). The procedure of RT-PCR amplification reaction is as follows: 40 cycles of 95°C for 10 s, 60°C for 30 s, and 72°C for 15 s with the primer sequences (Table 1), and GAPDH acted as an internal control. The relative messenger ribonucleic acid (mRNA) expression was calculated by the 2^ΔΔCT^ method.

**Table 1.** Sequences of Primers Used in Quantitative Real-Time Polymerase Chain Reaction.

Gene	Forward primer	Reverse primer
Bax	5′-GGCCCTTTTGCTTCAGGGTT-3′	5′-GGAAAAAGACCTCTCGGGGG-3′
Bcl-2	5′-CTTTGAGTTCGGTGGGGTCA-3′	5′-GGGCCGTACAGTTCCACAAA-3′
COX-2	5′-TTTGCATTCTTTGCCCGC-3′	5′-GGGAGGATACATCTCTCCATCAAT-3′
Cleaved caspase-3	5′-AGCAATAAATGAATGGGCTGAG-3′	5′-GTATGGAGAAATGGGCTGTAGG-3′
GAPDH	5′-GAACGGGAAGCTCACTGGC-3′	5′-GCATGTCAGATCCACAACGG-3′

Bax: BCL-2 associated X protein; Bcl-2: B-cell lymphoma-2; COX-2: cyclo-oxygen-ase-2; GAPDH: glyceraldehyde 3-phosphate dehydrogenase.

### Statistical Analysis

All experiments were carried out three times and the results were expressed as mean ± SD. All results obtained were analyzed using a one-way analysis of variance followed by Tukey’s multiple comparison test. Values with *P* < 0.05 were used to determine significant differences.

## Results

### Chemical Analysis of GLP and Its Degraded Fragments

**Fig. 1.**
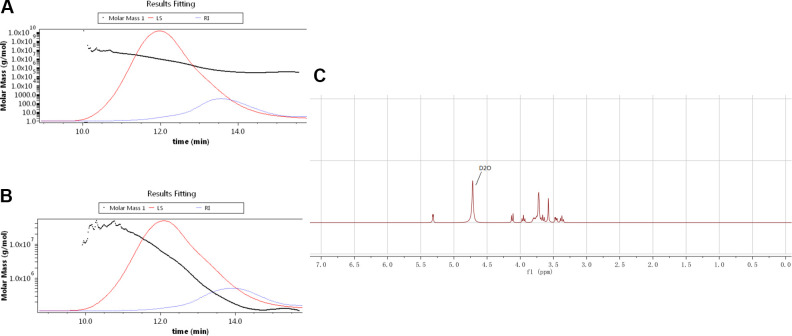
The molar mass, RI, and LS chromatograms for GLP (A) and EGLP (B). ^1^H-NMR spectra of EGLP (C). EGLP: enzymatically hydrolyzed *G. lucidum* polysaccharide; GLP: *G. lucidum* polysaccharide; LS: xxx; NMR: xxx; RI: xxx.

As displayed in Table 2, comparison of enzymatic degradation fractions showed no obvious difference existed but in molecular weight. The total polysaccharides contents of GLP and EGLP assayed by the phenol–sulfuric acid method were 88.63% and 89.16%, respectively. Meanwhile, the molecular weights of GLP and EGLP were determined by SEC-MALLS-RI (Fig. 1A, B). The molecular weight of GLP was 1,626 kDa, which was higher than EGLP (816.1 kDa). These results indicated that GLP was enzymatically degraded into lower molecular weight polysaccharide. In addition, Fig. 1C shows the ^1^H-NMR of EGLP. The typical peak distribution of polysaccharides was at *δ*H 3.0–5.5 ppm.

**Table 2.** The Chemical Property of Polysaccharide Fractions from *Ganoderma lucidum*.

Samples	GLP	EGLP
Mw (kDa)	1,626	816.1
Polydispersity (Mw/Mn)	3.65	4.06
Total polysaccharide (%)	88.63 ± 1.82	89.16 ± 1.49
Protein (%)	0.61 ± 0.02	0.47 ± 0.03
Uronic acids (%)	2.05 ± 0.11	2.62 ± 0.09
Sulfate (%)	3.14 ± 0.05	6.27 ± 0.06

EGLP: enzymatically hydrolyzed *G. lucidum* polysaccharide; GLP: *G. lucidum* polysaccharide; Mw: xxx; Mn: xxx.

### Effects of EGLP on the Viability of HCT-116 and CCD18-Co Cells

**Fig. 2.**
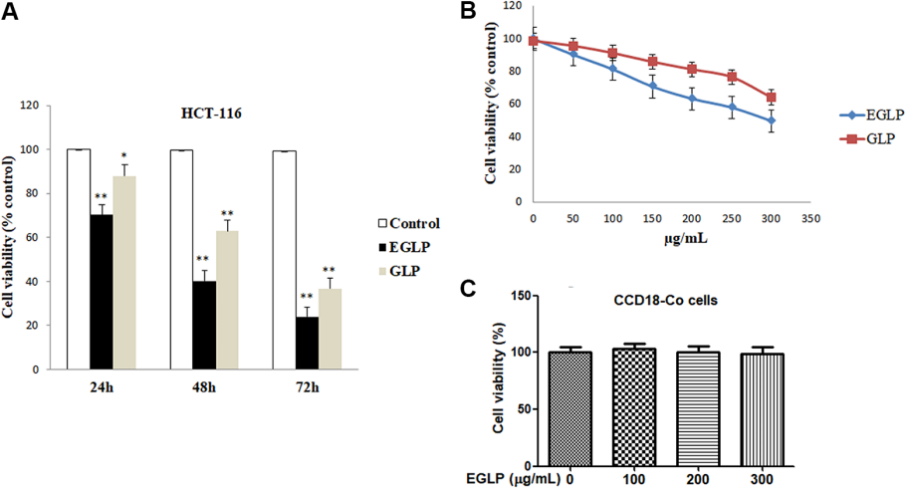
Inhibitory effects of native (GLP) and enzymatically modified (EGLP) polysaccharides from *Ganoderma lucidum* against the formation of colon cancer cells HCT-116. (A) HCT-116 cells were treated with EGLP or GLP (150 µg/ml) for 24, 48, and 72 h. (B) HCT-116 cells were treated with EGLP or GLP at different concentrations (0, 50, 100, 150, 200, 250, and 300 µg/ml) for 24 h. (C) CCD18-Co cells were treated with EGLP at different concentrations (0, 100, 200, and 300 µg/ml) for 24 h. The data are reported as mean ± SD (three independent experiments). **P < 0.01 (vs. the control group), *P < 0.05 (vs. the control group). CCD18-Co: xxx; EGLP: enzymatically hydrolyzed *G. lucidum* polysaccharide; GLP: *G. lucidum* polysaccharide; HCT-116: xxx.

The colon cancer cytotoxicity induced by GLP or EGLP was investigated in human colon cancer cells (HCT-116) using the MTT assay. As displayed in Fig. 2A, a time-dependent reduction in colon cancer cell viability was observed following treatment with the same concentration of GLP or EGLP in HCT-116 cells. Additionally, as shown in Fig. 2B, the proliferation of HCT-116 cells was dose-dependently suppressed by 50, 100, 150, 200, 250, and 300 µg/ml GLP or EGLP. Interestingly, the HCT-116 cells inhibitory effects of GLP were always lower than those of the enzymatic hydrolysates at the same incubation time. The results revealed that enzymatic degradation fractions of GLP exhibited more effective colon cancer cells inhibitory effect than did the intact GLP. This finding was consistent with the previous researches that the low molecular weight of degradation polysaccharides exerted stronger anticolon activity than the native polysaccharides^13,19^. To further investigate the effect of EGLP on the viability of normal cell line, CCD18-Co cells were treated with EGLP at different doses. As shown in Fig. 2C, the viability of CCD18-Co cells was not suppressed after treatment with EGLP. The findings showed that EGLP inhibited the viability of HCT-116 but not that of the normal cells in a concentration-dependent manner.

### EGLP Induces the Apoptosis of HCT-116 Cells

**Fig. 3.**
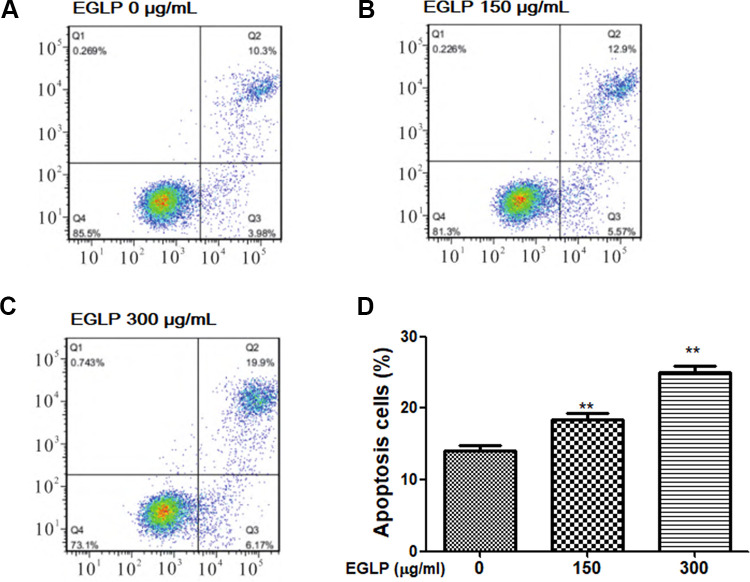
EGLP induces the apoptosis of HCT-116 cells. (A–C) HCT-116 cells were treated with EGLP (0, 150, and 300 µg/ml) for 24 h, and the apoptosis of HCT-116 cells was measured by flow cytometry. (D) The apoptosis cells were quantified. The data are reported as mean ± SD (three independent experiments). **P < 0.01 (vs. the control group). EGLP: enzymatically hydrolyzed *Ganoderma lucidum* polysaccharide; HCT-116: xxx.

The effects of EGLP on HCT-116 cells apoptosis were measured by Annexin V/PI staining. As shown in Fig. 3, the apoptosis rate was increased upon EGLP treatment in HCT-116 cells. Additionally, the total apoptotic cell populations were up to 26.07% when compared to the control group (*P* < 0.01). These results indicated that the suppression of HCT-116 cell growth by EGLP is related to apoptosis.

### EGLP Regulated the Expression of Cell Apoptosis-Related Proteins in HCT-116 Cells

**Fig. 4.**
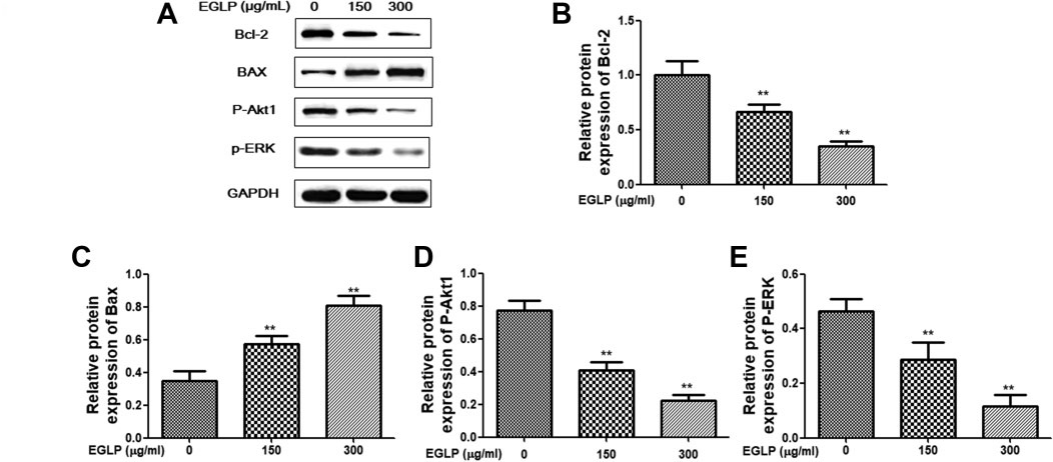
Effect of EGLP in the apoptotic pathway. HCT-116 cells were treated with EGLP (150 and 300 µg/ml) for 24 h. (A) Western immunoblots of prepared protein and densitometry analysis of protein expression: (B) Bcl-2, (C) Bax, (D) P-Akt1, (E) P-ERK. The results are reported as mean ± SD (three independent experiments). **P < 0.01 (vs. the control group). Bax: xxx; Bcl-2: xxx; EGLP: enzymatically hydrolyzed *Ganoderma lucidum* polysaccharide; HCT-116: xxx; P-Akt1: xxx; P-ERK: xxx.

In the present study, western blotting was performed to investigate the underlying mechanism by which EGLP induced cell apoptosis. As shown in Fig. 4, our findings indicated that the protein expressions of Bcl-2, P-ERK, and P-Akt1, which inhibit the cell apoptosis, were decreased in an EGLP dose-dependent manner. Additionally, the cell apoptosis progressive proteins (Bax) were increased in EGLP dose-dependent manner.

### EGLP Regulated the Expression of Cell Apoptosis-Related mRNA in HCT-116 Cells

**Fig. 5.**
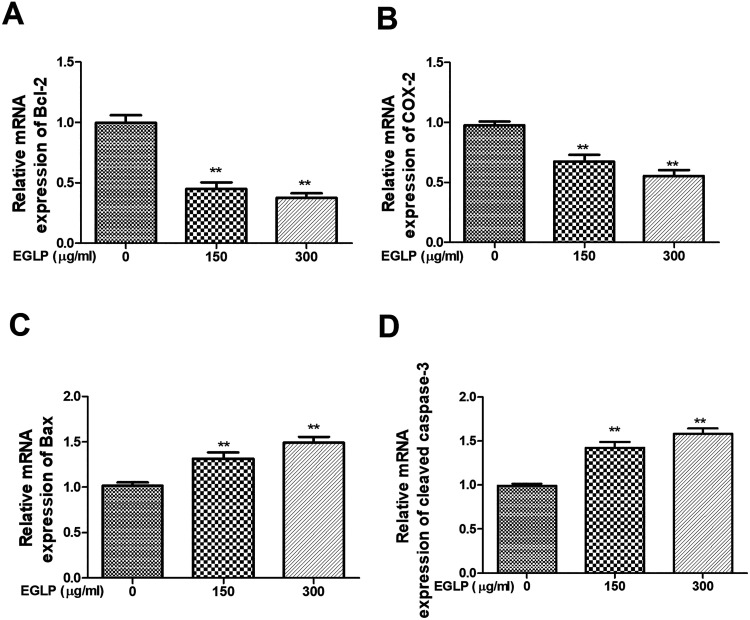
The mRNA expression levels of Bcl-2 (A), COX-2 (B), Bax (C), and cleaved caspase-3 (D) were measured by qRT-PCR assay. The results are reported as mean ± SD (three independent experiments). **P < 0.01 (vs. the control group). Bax: xxx; Bcl-2: xxx; COX-2: xxx; EGLP: enzymatically hydrolyzed *Ganoderma lucidum* polysaccharide; mRNA: xxx; qRT-PCR: quantitative real-time polymerase chain reaction.

As shown in Fig. 5, our findings indicated that the mRNA expressions of Bcl-2 and COX-2, which inhibit the cell apoptosis, were decreased in an EGLP dose-dependent manner. Additionally, the mRNA expressions of Bax and cleaved caspase-3, which progress the cell apoptosis, were increased in an EGLP dose-dependent manner.

## Discussion

Colorectal cancer is one of the most common adenocarcinomas and the third leading cause of tumor-associated incidence and mortality in the world^20^. At present, the principal treatment of colorectal cancer usually includes chemotherapy and surgical resection. However, organ toxicity and limited effectiveness remain obstacles to the use of those therapies. Therefore, developing a novel tumor inhibitor with low toxicity and high efficiency has become the focus of the present research. Previous reports have indicated that polysaccharides exert effective antitumor effect and broad spectrum^21^. However, detailed mechanisms and related regulatory aspects remain unclear in colorectal cancer treatment. In our study, the findings indicated that EGLP suppresses the viability of HCT-116 cells in time- and concentration-dependent manner. Besides, EGLP also induces HCT-116 cells apoptosis.

Apoptosis is a general phenomenon in cytotoxicity evoked by antitumor drugs and it is an important mechanism involved in cell death, growth, invasion, and differentiation. Additionally, cell apoptosis is concerned with the malignant tumor progression^22^. Bcl-2 and Bax are commonly considered as important mediators of cell apoptosis and belong to Bcl-2 families^23^. A previous report has indicated that the mechanism of *Rhizopus nigricans* polysaccharide in the promotion of apoptosis in colorectal cancer might be associated with the regulation of the Bax/Bcl-2 signaling pathway^24^. Additionally, Bax activation and Bcl-2 suppression are considered as a promising therapy for tumor treatment^25,26^. The previous study has indicated that the improvement in triggering of the mitochondrial pathway by cyclophosphamide might be associated with the effects of GLP on the regulation of Bcl-2 family proteins^27^, which reminded us to speculate that EGLP may promote HCT-116 cells apoptosis via the regulation of Bax/Bcl-2 protein expression. Lots of reports have indicated that both mitogen-activated protein kinase/extracellular regulated protein kinases (MAPK/ERK) and phosphatidylinositol -3- hydroxykinase (PI3K/Akt) are representative signaling pathways and have been previously indicated to be activated in various types of tumors or cells, including colorectal cancer^28,29^. Besides, the overexpression of the PI3K/Akt signaling pathway facilitates the depolymerization of Bcl-xl and Bcl-2, resulting in the inactivation of Caspase-9 and Caspase-3, and finally leads to the growth and proliferation of cancer cells^29^. An accumulation of reports indicated that the activation of COX-2 in various types of tumors is related to the progression of the malignant tumor. Additionally, the COX-2 inhibitor could inhibit the growth of cancer cells and improve the antiangiogenic tumor therapy^22^. In our research, we observed that EGLP evoked apoptosis in HCT-116 cells. More importantly, the expressions of COX-2, Bcl-2, P-ERK, and p-Akt1 were decreased, and the expressions of Bax and cleaved caspase-3 were increased after EGLP treatment. Previous reports indicated that GLP promotes the apoptosis of Human premortem leukemia cells (HL)-60 cells via regulating the expression of Bax, Bcl-2, and cleaved caspase-3. And enzymatically hydrolyzed *G. lucidum* crude polysaccharide possesses prior anticervical cancer activity than the crude polysaccharide^30,31^. The previous findings were in accordance with our results. Our results implied that EGLP might promote colon cancer cells apoptosis via the regulation of p-Akt1/P-ERK and Bax/Bcl-2 protein expression.

## Conclusions

In conclusion, the present study showed for the first time that EGLP was capable of inhibiting the growth of HCT-116 cells by promoting apoptosis via the Akt/ERK signaling pathway. Our results provided the scientific evidence for further development of EGLP as a novel antitumor agent for colon cancer treatment.
